# Cryo-derived plants through embryogenesis showed same levels of vinblastine and vincristine (anticancer) in *Catharanthus roseus* and had normal genome size

**DOI:** 10.1038/s41598-022-20993-z

**Published:** 2022-10-05

**Authors:** A. Mujib, Samar Fatima, Moien Qadir Malik

**Affiliations:** Cellular Differentiation and Molecular Genetics Section, Department of Botany, Jamia Hamdard, New Delhi, India

**Keywords:** Biological techniques, Genetics

## Abstract

Cryopreservation of rare plant materials is an important approach for preserving germplasms and is a good added concept to tissue banking. The preservation of embryogenic cell suspensions is even more valuable as the tissues facilitate in producing millions of embryos, plantlets and generates transgenics en masse. *Catharanthus roseus* is a medicinally important plant that produces a variety of anticancerous phytocompounds and needs conservation of alkaloid producing cell lines. In this study, embryogenic tissue banking has been attempted in *C. roseus* by the two-step cryopreservation method combining cryoprotection and dehydration. Prior to plunging into liquid nitrogen (LN), the tissues were exposed to osmotic—and cryoprotective agents. Two osmotic agents (sugar and sorbitol) and three cryoprotective compounds, polyethylene glycol (PEG), dimethyl sulfoxide (DMSO) and glycerol were used at varying concentrations to protect cells from freezing damages. Both sucrose and sorbitol increased callus biomass post-cryopreservation; the influence of sucrose was however, more prominent. Embryogenic tissue treated in medium with 0.4 M sucrose for 2 days followed by 5% PEG for 2 h showed maximum viability before (83%) and after (55%) cryopreservation, high regrowth percentage (77%) and produced an average 9 cell colonies per Petri dish. Additionally, dehydration (1–5 h) was tested to reduce water content for improving viability and regrowth of cryopreserved embryogenic cells. Among the various tested cryoprotective conditions, the highest (72%) viability was observed following the combination of treatments with 0.4 M sucrose (2 days),10% PEG (2 h) and dehydration (2 h). Maximum regrowth percentage (88%) and 12 colonies/petri dish was noted in combination of 0.4 M sucrose + 5% PEG. The cryopreserved calli differentiated into somatic embryos (52.78–54.33 globular embryos/callus mass) in NAA (0.5 mg/l) and BAP (0.5–1.0 mg/l) added media. Plantlets were successfully regenerated from cryopreserved tissue and the 2C DNA was estimated through flow cytometry. The genome size of cryopreserved regenerant was 1.51 pg/2C, which is similar to field-grown *Catharanthus* plants. Vinblastine and vincristine levels were nearly the same in mother plant’s and frozen (cryopreserved) leaf tissue. The post cryopreservation embryogenesis protocol may be used for continuous production of plants for future applications.

## Introduction

*Catharanthus roseus* L. (G.) Don is commonly known as Madagascar periwinkle. This ornamental, medicinal plant belongs to the family Apocynaceae. The plant is distributed throughout the tropics but is naturalized in America, Africa, Australia, the Southern part of Europe, the Pacific Ocean islands and in India. The plant has historically been used to treat a wide range of diseases like diabetes, as an astringent, diuretic and cough remedy^1^. The two indolic alkaloids vinblastine and vincristine are used in chemotherapy against leukemias, Hodgkin's disease and solid tumors^2,3^, but the yield is unfortunately very low (0.0005% dry weight basis). As an alternative, the cell cultures especially the embryogenic tissues have been employed in alkaloid enrichment programme^4–6^. During in vitro embryogenesis, the embryogenic cells generate somatic embryos following a series of morphological, biochemical and molecular alterations^7^. Embryogenic cultures have a wide range of biotechnological applications in areas like artificial seeds production, micropropagation, transgenic development and cryopreservation^8^.

Cryopreservation has been practiced for a long time^8,9^ using in vivo grown plant parts like zygotic embryos and seeds^10,11^ and in vitro derived tissues like anthers, buds and somatic embryos^12^. Various methods like encapsulation-dehydration, vitrification, encapsulation-vitrification, droplet-vitrification have been employed for preserving tissues^9,13,14^. In these protocols, the osmotic potential is maintained with sugars or sugar alcohols in medium. These osmotic agents provide energy to the growing tissues and create osmotic stress^15,16^. Ultra-low temperature using liquid nitrogen ceases cellular metabolic functions partially and stores plant materials indefinitely; on a suitable medium following thawing, the cells restore growth without any genetic change^16,17^. The success is, however, depends upon dehydration of tissue water as crystallization promotes freezing injuries to cells. Shatnawi et al.^14^ used dehydration by placing the tissue, cell suspension in laminar airflow cabinet for varying periods until the water level comes to 30% or below before plunged into liquid nitrogen. The de-differentiated embryogenic tissues are vulnerable to low temperature as these are soft and simple without much protective layer, which need cryogenic protection^17^. The most method uses rapid cooling or plunging of tissues into liquid nitrogen, while other methods require a cryoprotective treatment and cooling^17,18^. A variety of cryoprotective agents like glycerol, polyethylene glycol (PEG), dimethyl sulfoxide(DMSO) have been used either alone or in mixture^19,20^. For in vitro cultures, the pre-cryopreservation medium contains sucrose or sorbitol as osmotic agents and cryoprotective compound like DMSO. In contrast, a typical post-cryopreservation medium devoids of these osmotic and cryoprotective agents^21^. During post-cryopreservation, the tissues are cultured briefly in medium to diffuse out osmotic and cryoprotective agents from tissues; later transferred to fresh medium without osmotic and cryoprotective agents. In non-embryogenic *Catharanthus* culture, the use of DMSO and sorbitol was noted to be efficient in preventing freezing in earlier observation^22–24^. The impact of dehydration in demonstrating efficiency and success of cryopreservation has never been tested in *C. roseus* since trace water level is crucial to tissue viability and regrowth. The importance of embryogenic tissue preservation has also not been realized fully as it is the unlimited source of embryos and plants, amenable to en-masse transgenic production. In the present study, the effectiveness of dehydration, the role of osmotic agents and their combinations with cryoprotective agents were assessed by monitoring the viability and regrowth of cryopreserved cells. The genome size (2C DNA content) of the plants regenerated from cryopreserved cell cultures and the level of main alkaloids, vinblastine and vincristine were measured and compared with field-grown plant as to check the genetic fidelity/homogeneity as cryopreservation involves diverse chemicals and liquid nitrogen, which produce severe cold shock or stress.

## Results

### The influence of osmo—and cryoprotectants on cell viability

The embryogenic cells were cultured separately in 0.2, 0.4 and 0.6 M sucrose and sorbitol added MS medium for 48 h and the viability was measured.Various compounds i.e.PEG, DMSO and glycerol were used as cryoprotectants (CP) either alone or in combinations. Treatment of the embryogenic suspension cultured in 0.2 M sucrose or sorbitol had no effect on viability after cryopreservation when combined with various CP; therefore,these treatments were discontinued. Combination of 5% PEG (CP-A) with 0.4 M sucrose was the most effective and showed 83.26% and 55.23% viability before and after cryopreservation, respectively (Table 1). Increase in PEG level (CP-B) reduced cell viability before (71.25%) and after (41.32%) cryopreservation. PEG in combination with DMSO and glycerol (CP-C to CP-F) was less efficient in improving cell viability. In 0.4 M sorbitol added 5% PEG medium, the maximum cell viability percentage (46.12%) was noted (Table 2).

**Table 1:** Percent viability of cell suspensions of *C. roseus* before and after cryopreservation in sucrose-containing media.

Cryoprotectant treatment	Sucrose concentration in medium (osmoprotectant treatment)				
	0.2 M sucrose	0.4 M sucrose		0.6 M sucrose	
	− LN	− LN	+ LN	− LN	+ LN
Control	98.50 ± 0.60a	97.48 ± 0.50a	0.0 ± 0.0f	87.74 ± 2.29a	0.0 ± 0.0g
5% PEG	85.03 ± 2.32b	83.26 ± 1.82b	55.23 ± 1.21a	76.12 ± 2.71b	43.41 ± 2.12a
10% PEG	74.62 ± 1.20c	71.25 ± 1.68c	41.32 ± 2.16b	63.51 ± 1.70c	37.12 ± 1.33b
5% PEG + 5% DMSO	59.76 ± 3.11e	59.06 ± 2.40d	38.25 ± 1.67c	53.18 ± 1.82d	32.42 ± 1.18c
5% PEG + 10% DMSO	45.33 ± 1.36f	41.98 ± 2.18f	27.54 ± 3.72d	35.11 ± 1.68g	28.12 ± 1.54d
5% PEG + 5% glycerol	63.01 ± 1.82d	59.32 ± 1.36d	28.25 ± 1.84d	48.91 ± 3.37e	23.65 ± 1.75e
5% PEG + 10% glycerol	48.87 ± 2.86f	46.52 ± 2.44e	23.25 ± 1.10e	39.84 ± 1.43f	18.21 ± 1.67f

Cell suspension was cultured in liquid medium with 0.2–0.6 M sucrose for 2 days (osmoprotectant treatment) followed by 2 h cryoprotecant treatment and two-step cryopreservation.

Absence of column (0.2 M/+ LN) corresponds to zero viability of cells in all cryopreserved treatments. Values are means ± standard errors of 3 replicates of two experiments. Mean values within a column followed by different letters are significantly different at p = 0.05 according to Duncan’s Multiple Range Test (DMRT).

*− LN* before cryopreservation, * + LN* after cryopreservation.

**Table 2:** Percent viability of cell suspensions of *C. roseus* before and after cryopreservation in sorbitol containing media.

Treatments	Viability %				
	0.2 M	0.4 M		0.6 M	
	− LN	− LN	+ LN	− LN	+ LN
Control	97.85 ± 0.29a	93.18 ± 0.75a	0.0 ± 0.0e	84.86 ± 0.23a	0.0 ± 0.0f
5% PEG	83.18 ± 3.20b	78.80 ± 2.18b	46.12 ± 1.81a	75.12 ± 2.71b	38.2 ± 1.32a
10% PEG	70.55 ± 1.88c	69.12 ± 1.16c	33.25 ± 2.66b	61.22 ± 2.17c	31.36 ± 2.30b
5% PEG + 5% DMSO	58.74 ± 2.18e	54.64 ± 2.14d	28.94 ± 1.54c	51.04 ± 1.14d	26.62 ± 2.21c
5% PEG + 10% DMSO	42.45 ± 3.12f	38.22 ± 2.70f	21.14 ± 2.17d	31.42 ± 2.18f	24.66 ± 1.42c
5% PEG + 5% glycerol	63.44 ± 2.18d	56.77 ± 2.12d	27.26 ± 1.40c	45.11 ± 2.61e	19.74 ± 1.50d
5% PEG + 10% glycerol	45.56 ± 3.17f	41.15 ± 1.46e	21.33 ± 1.21d	32.46 ± 2.14f	12.88 ± 2.16e

Cell suspension was cultured in liquid medium with 0.2–0.6 M sucrose for 2 days (osmoprotectant treatment) followed by 2 h cryoprotecant treatment and two-step cryopreservation.

Absence of column (0.2 M/ + LN) corresponds to zero viability of cells in all cryopreserved treatments. Values are means ± standard errors of 3 replicates of two experiments.Mean values within a column followed by different letters are significantly different at p = 0.05 according to Duncan’s Multiple Range Test (DMRT).

*− LN* before cryopreservation, *+ LN* after cryopreservation.

### Growth after cryopreservation

The most important criterion for evaluating the effectiveness of osmotica and the cryoprotective agents was the regrowth of cells following plating on an optimized medium. Regrowth was observed in all treatments with sugar levels ≥ 0.4 M. The lower levels failed to protect cells from severe ultra-low temperature impact.

**Figure 1:**
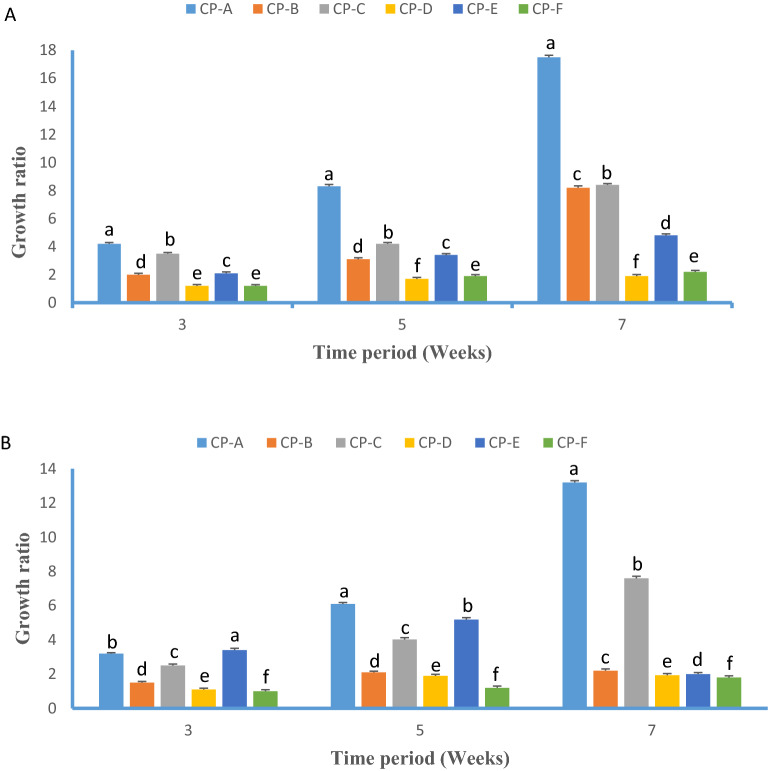
Growth ratio (W_o_/W_i_) of cryopreserved embryogenic cell suspension osmoprotected in (**A**) 0.4 M sucrose and (**B**) 0.6 M sucrose added MS medium (1.0 mg/l NAA and 1.5 mg/l BA) recorded 3, 5 and 7 weeks after rewarming. *W*_*o*_ initial fresh weight and *W*_*i*_ final fresh weight, respectively. CP abbreviations are CP-A: 5% PEG, CP-B: 10% PEG, CP-C: 5% PEG + 5% DMSO, CP-D: 5% PEG + 10% DMSO, CP-E: 5% PEG + 5% glycerol, CP-F: 5% PEG + 10% glycerol.

**Figure 2:**
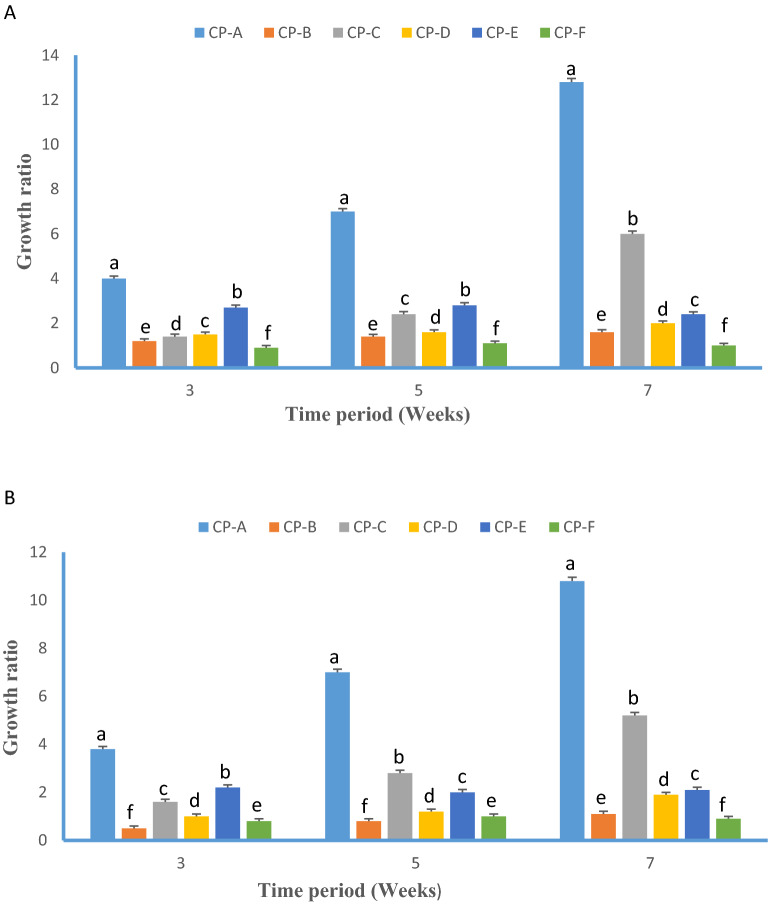
Growth ratio (W_o_/W_i_) of cryopreserved embryogenic cell suspension osmoprotected in (**A**) 0.4 M and (**B**) 0.6 M sorbitol added MS medium (1.0 mg/l NAA and 1.5 mg/l BA) recorded 3, 5 and 7 weeks after rewarming. *W*_*o*_ initial fresh weight and *W*i final fresh weight, respectively. CP abbreviations are CP-A: 5% PEG, CP-B: 10% PEG, CP-C: 5% PEG + 5% DMSO, CP-D: 5% PEG + 10% DMSO, CP-E: 5% PEG + 5% glycerol, CP-F: 5% PEG + 10% glycerol.

The growth ratio (W_0_/Wi) of callus was monitored after 3, 5 and 7 weeks of culture on solid MS. Cells cryopreserved after osmoprotection in 0.4 M sucrose containing medium (Fig. 1A) showed faster growth compared to osmoprotection with 0.6 M sucrose (Fig. 1B). Among cryoprotectant treatments, good growth was achieved on 5% PEG with a growth ratio of 8.45 and 16.54 at 5 and 7 weeks of culture (CP-A), followed by10% PEG (CP-B). All PEG combinations with DMSO and glycerol resulted in very poor growth compared to PEG alone treatments CP-D, CP-E and CP-F (Fig. 1A). Osmoprotection with 0.4 M sorbitol resulted in faster growth compared to 0.6 M sorbitol (Fig. 2A,B). In 5% PEG treatment (CP-A), the embryogenic cell culture osmoprotected with 0.4 M sorbitol showed 7.33and 12.86 growth ratio at 5 and 7 weeks of culture, which is little better than for the 0.6 M sorbitol added CP-A (6.98 and 10.45 growth ratio). In all cases, the growth during the first 5 weeks was poor but intensified within 5–7 weeks. In summary, based on cell viability and growth, sucrose worked better than sorbitol for osmoprotection,with 0.4 M been optimal for both compounds. Among cryoprotectants, 5% or 10% PEG (CP-A and CP-B) were the most efficient, followed by 5% PEG + 5% DMSO(CP-C) and 5% PEG + 5% glycerol (CP-E). The highest regrowth percentage (77.7%) with a 9.26 mean number of cell colonies per Petri dish was noted for cell culture cryopreserved after osmoprotection in 0.4 M sucrose containing medium and 5% PEG cryoprotection (CP-A) (Table 3).

**Table 3:** Regrowth of *C. roseus* embryogenic cultures cryopreserved following different osmoprotection and cryoprotection treatments.

Osmoprotectant	Cryoprotectant	Number of Petri dishes with regrowth/regrowth %	Mean number of cell colonies grown per Petri dish
0.4 M sucrose	CP-A	7/77.7a	9.26 ± 1.01a
	CP-B	2/22.2c	3.11 ± 0.18b
	CP-C	3/33.3b	3.67 ± 1.12b
	CP-D	1/11.1d	1.29 ± 0.42d
	CP-E	3/33.3b	2.72 ± 0.08c
	CP-F	2/22.2c	2.15 ± 0.18c
0.6 M sucrose	CP-A	5/55.5a	7.18 ± 1.21a
	CP-B	2/22.2c	2.16 ± 0.44c
	CP-C	3/33.3b	3.10 ± 1.11b
	CP-D	2/22.2c	2.12 ± 0.26c
	CP-E	3/33.3b	3.68 ± 0.61b
	CP-F	1/11.1d	0.95 ± 0.01d
0.4 M sorbitol	CP-A	5/55.5a	5.83 ± 1.32a
	CP-B	3/33.3c	3.14 ± 1.10c
	CP-C	2/22.2d	2.10 ± 0.22c
	CP-D	2/22.2d	2.06 ± 0.16c
	CP-E	4/44.4b	4.11 ± 1.14b
	CP-F	2/22.2d	1.22 ± 0.18d
0.6 M sorbitol	CP-A	3/33.3a	1.65 ± 0.13a
	CP-B	1/11.1c	0.26 ± 0.05e
	CP-C	2/22.2b	1.12 ± 0.08b
	CP-D	1/11.1c	0.53 ± 0.11d
	CP-E	2/22.2b	1.12 ± 0.20b
	CP-F	1/11.1c	0.66 ± 0.05c

Data were recorded 4 weeks after rewarming.

Nine Petri dishes were used and the number of Petri dishes showing cell regrowth was recorded.

Values are means ± standard errors of 3 replicates of two experiments. Mean values within a column followed by different letters are significantly different at p = 0.05 according to Duncan’s Multiple Range Test (DMRT).

### Influence of dehydration on cell viability and regrowth

**Figure 3:**
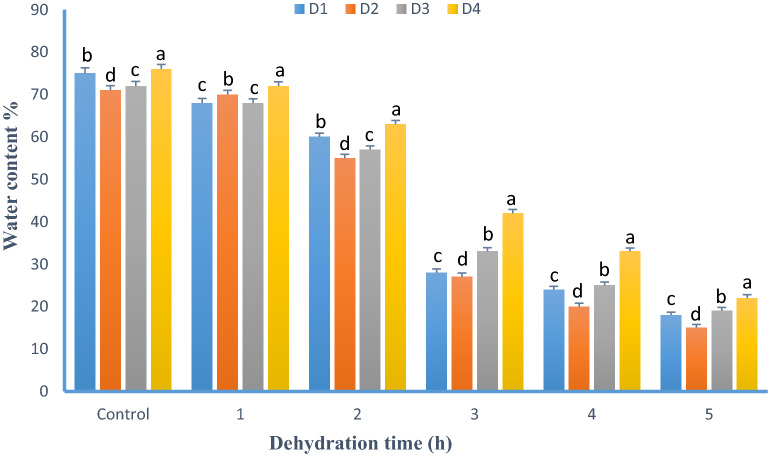
Effect of dehydration (1–5 h) on water content of cryoprotected *C. roseus* suspension cells. The following treatments and code were used: 0.4 M sucrose + 5% DMSO (D1); 0.4 M sucrose + 5% DMSO + 5% glycerol (D2); 0.4 M sorbitol, 5% DMSO + 5% glycerol (D3); 0.4 M sucrose + 5% PEG (D4).

The best cryoprotectant combinations selected at previous steps were used to test the effect of cell dehydration before cryopreservation. The alteration of the water level of the select suspension cells was measured hourly (Fig. 3). In addition, cell viability was recorded for CP-A and CP-B treatments. In 5% PEG treatment, the initial water content was 74.10% (fresh weight basis); it rapidly decreased to 31.14% after 3 h and dropped to 18.74% after 5 h of dehydration. Similar water loss was observed in other treatments. In 5% PEG, the viability was 74.85% at control (0 h) which decreased to 71.34 and 39.55% after 3 and 5 h dehydration respectively as the water level dropped. In 10% PEG, the highest viability (72.17%) was observed after 2 h of dehydration (Table 4).

**Table 4:** Effect of dehydration (1-5 h) on viability (%) of cryoprotected cells before and after cryopreservation.

Dehydration time (h)	Viability %			
	5% PEG(CP-A)		10% PEG (CP-B)	
	− LN	+ LN	− LN	+ LN
0	74.85 ± 2.03a	63.59 ± 2.57c	74.70 ± 2.01a	69.54 ± 0.39c
1	71.89 ± 1.16b	66.44 ± 2.11b	74.43 ± 1.10a	70.18 ± 1.86b
2	72.76 ± 0.98b	69.86 ± 1.85a	73.66 ± 2.11b	72.17 ± 1.53a
3	71.34 ± 1.21b	71.10 ± 1.32a	70.75 ± 3.61c	69.84 ± 0.82c
4	54.44 ± 2.39c	47.32 ± 1.21d	52.38 ± 2.18d	48.52 ± 2.76d
5	39.55 ± 2.04d	23.11 ± 2.03e	36.25 ± 1.63e	22.17 ± 1.12e

Cell suspension was cultured in liquid medium with 0.2–0.6 M sucrose for 2 days (osmoprotectant treatment) followed by 2 h cryoprotecant treatment and two-step cryopreservation.

Values are means ± standard errors of 3 replicates of two experiments. Mean values within a column followed by different letters are significantly different at p = 0.05 according to Duncan’s Multiple Range Test (DMRT).

*− LN* before cryopreservation, *+ LN* after cryopreservation.

Following dehydration, the cells were placed on a solid medium for regrowth and the mean number of cell colonies was counted. The maximum regrowth percentage (88.8%) and the mean number of regrown colonies/Petri dish (12.65) was obtained in 5% PEG treatment (Table 5). Other treatments (CP-B and CP-E) also exhibited quite a high regrowth percentage and produced 6.73–9.68 numbers of colonies/petri dish. Thus, 2 h dehydration in 5% PEG treatment was noted to be the efficient conditions for cell viability and regrowth process.

**Table 5:** Optimized cryopreservation procedure (cryoprotectant treatments, dehydration time) and regrowth of cryopreserved *C. roseus* culture.

Cryoprotectant treatment	Dehydration time (h)	Number of petri dishes with regrowth %	Mean number of cell colonies/petri dish
CP-A	2	8/88.8a	12.65 ± 1.07a
CP-B	2	7/77.7b	7.78 ± 0.65c
CP-C	2	6/66.6c	9.68 ± 1.67b
CP-E	2	7/77.7b	6.73 ± 0.43d

Nine Petri dishes were used in which the number of Petri dishes showing regrowth was counted. 0.4 M Sucrose and the mentioned CP agents were also used (CP-A: 5% PEG, CP-B: 10% PEG, CP-C: 5% PEG + 5% DMSO, CP-E: 5% PEG + 5% glycerol).Values are means ± standard errors of 3 replicates of two experiments. Mean values within a column followed by different letters are significantly different at p = 0.05 according to Duncan’s Multiple Range Test (DMRT).

### Embryo differentiation from regrown callus

The colonies grew well in medium and the calli sizes increased with every sub-culturing. The differentiation of somatic embryos from regrown callus were again started in NAA and BAP added media. The numbers of embryos at different developing stages were different but close to non-frozen culture, for example, the total numbers of cotyledonary embryos in control condition is 6.25, in CP-A, it is 6.61; and in CP-B, it is 5.38 (Table 6). No difference in embryo morphology was noted between cryopreserved and non- cryopreserved cultures.

**Table 6:** Number of embryos at different developing stages in optimized cryopreservation procedure (cryoprotectant treatments with 2 h dehydration time) in *C. roseus* cell culture.

Cryoprotectant treatment	Embryo development stages			
	Globular	Heart	Torpedo	Cotyledonary
Control	61.5 ± 1.18a	22.5 ± 1.2a	9.00 ± 0.80c	6.25 ± 1.7a
5% PEG (CP-A)	54.33 ± 2.27b	21.11 ± 2.34b	14.34 ± 1.34a	6.61 ± 0.89a
10% PEG (CP-B)	52.78 ± 1.85c	19.23 ± 1.67c	12.55 ± 1.67b	5.38 ± 1.54b

Optimized MS medium contained NAA (0.5 mg/l) + BAP (1.5 mg/l). Values are means ± standard errors of 3 replicates of two experiments. Mean values within a column followed by different letters are significantly different at p = 0.05 according to Duncan’s Multiple Range Test (DMRT).

### Plant regeneration and 2C DNA content of cryopreserved plant

**Figure 4:**
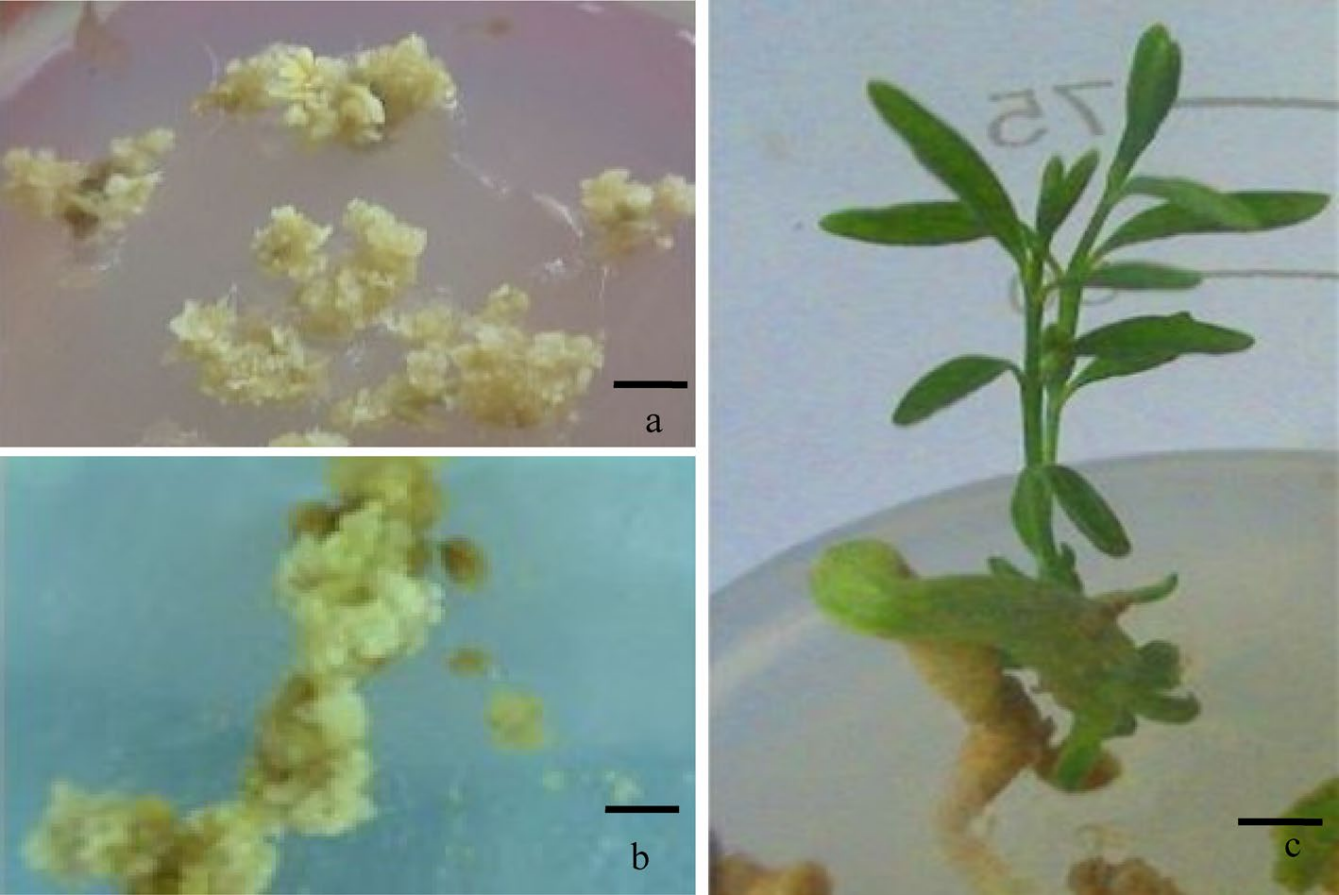
(**a**) Dehydrated culture producing callus (4 weeks after rewarming). (**b**) Further growth of callus developed from cryopreserved cells in optimized medium (6 weeks old). (**c**) In vitro plant regenerated from cryopreserved embryogenic callus (bar **a**–**b** 0.5 cm; **c** 1 cm).

**Figure 5:**
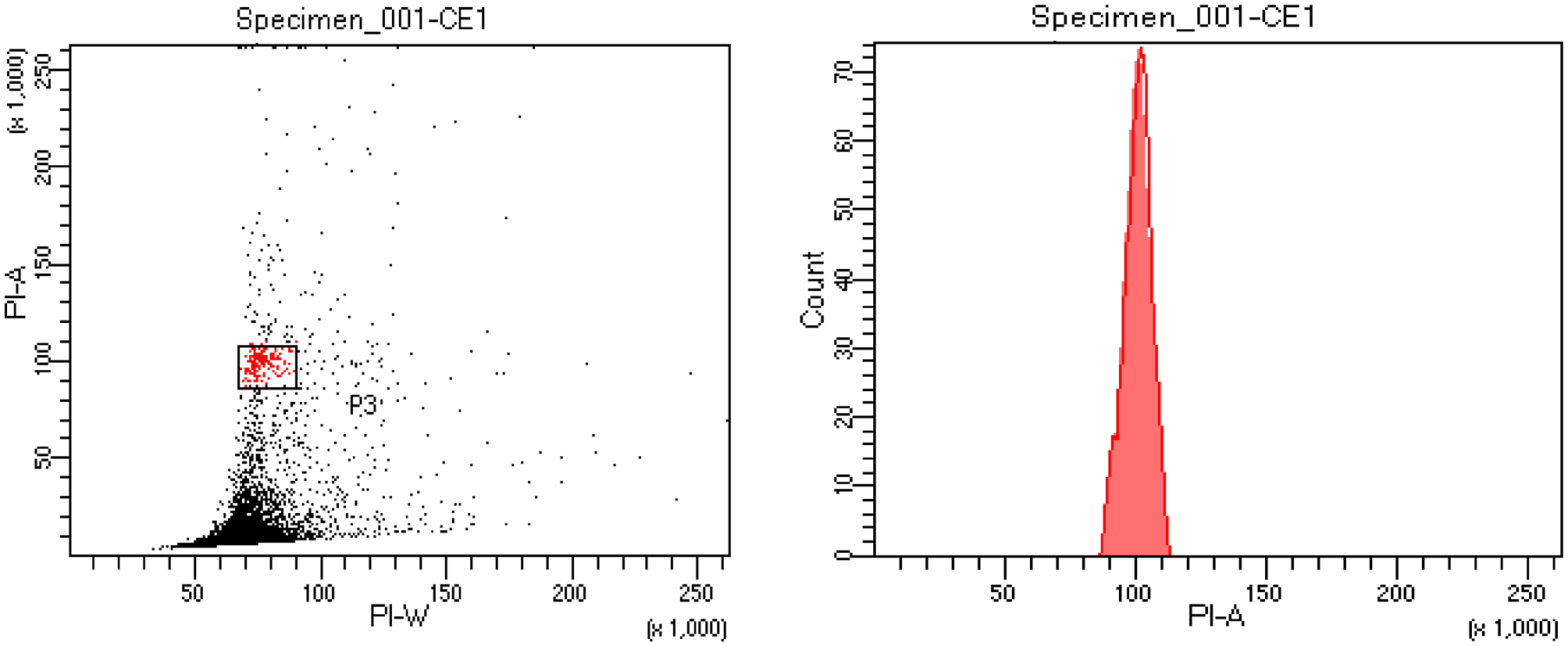
Dot plot and histogram of relative 2C DNA content of nuclei isolated from leaves of *C. roseus* plants regenerated from cryopreserved embryogenic cell culture.

Plantlets were regenerated from cryopreserved embryogenic cells (Fig. 4a–c) via embryos. The process of obtaining plants (‘callus to embryo to plant’) followed the same route through maturation and germination of embryos.The plantlet recovery time (embryo to plant) was also the same (4–5 months) between cryopreserved and non-cryopreserved cultures (control). The genome size of embryo regenerated plant was measured by flow cytometry. The histogram of 2C DNA content (Fig. 5) of cryoderived plant is 1.51 pg which is similar to field-grown *Catharanthus* plant. The DNA level of the cryo-derived plant is thus stable, showing identical genome size.

### Alkaloid yield in control and leaf of plants developed from cryopreserved embryogenic culture

The leaf of mother plant (control) and cryopreserved derived plants was harvested and the yield of vinblastine and vincristine was measured as this tissue was reported to be the primary source of alkaloids. The comparative yield of vinblastine and vincristine of above two sources is presented in Table 7. The vinblastine content of control leaf is 13.10 µgm gm^−1^ DW, while the level was 12.96 µgm gm^-1^ DW in leaf derived from cryopreserved tissue; the yield difference was very less. Similarly the vincristine yield was nearly the same when the two sources of leaves were compared.

**Table 7:** Vinblastine and vincristine yield (µgm gm^-1^ DW) in control plants and plants developed from cryopreserved embryogenic cell culture.

Tissue used	Vinblastine	Vincristine
Leaf of control plants	13.10 ± 0.06a	4.22 ± 0.03a
Leaf of plants developed from cryopreserved embryogenic culture	12.96 ± 0.05b	3.99 ± 0.03b

Values are means ± standard errors of 3 replicates of two experiments (n = 6); within each column means followed by the same letter are not significantly different at p = 0.05 according to DMRT.

## Discussion

The level of cellular water content and their translocation across cells regulate cryopreservation success in a major way. In this present investigation, a cryoprotection and dehydration based cryopreservation protocol of *C. roseus* has been established by utilizing osmotic and cryoprotective agents; and the plant regeneration was achieved. In this study, different osmotic agents, cryoprotectants and tissue dehydration were used with fair success. These compounds collectively regulated hydration of cells and prevent cellular injuries^10^. The treatment of tissues (embryogenic cell, somatic embryo or dormant bud) with elevated level of sugar, abscisic acid (ABA) increase the survivability of cells by improving desiccation-adaptive ability^25^. The water level and its movement across the cell membrane primarily determine the success of cryopreservation. The water level needs to be lowered by using various cryoprotective agents and osmotica^26^. Rapid plunging of tissues in liquid nitrogen temporarily ceases physiological function and when placed on optimum cultural regrowth conditions after thawing, the cells resume normal growth. The incorporation of sugar or sugar alcohol osmoticum protects cells from obvious damages by providing extra energy^10^. Huebinger^27^ noted that the DMSO and PEG have a direct influence on lipid mobility in plasma membrane as the membrane properties are regulated by varied sterol content. Several physiological, biochemical and molecular modifications have been noted in *Arabidopsis* cell wall in response to cold adaptation to increased freeze tolerance^28^. Desiccation—a simple process used to avoid crystallization of tissue water as enhanced levels of osmotica and dehydration cause cytosol water to vitrify^29^; however, the potential of it is still untapped. In this study, the suspended cells of *C. roseus* were treated with various concentrations of osmotica and cryoprotective agents, the moderate level was noted to be more efficient in promoting viability and regrowth than the higher levels, very consistent with earlier observation^30,31^. Similarly earlier research indicated that the plant vitrification solution 2 (PVS2)—a mixture of compounds containing 30% (w/v) glycerol + 15% (w/v) PEG + 15% (w/v) DMSO + 0.4 M sucrose (osmoticum) added medium improved preservation of cells and promoted culture regrowth^19,32^. The extra diffusion/removal of water, however, damages the prospect of survivability and in such a situation, the slow gradual cooling and inclusion of cryoprotective agents like DMSO have been noted to be successful in several investigated plants genera^33^. In this study, we noted that the cell viability percentage is quite high in PEG amended medium, which suggests its immense important role in protecting and reviving cells in standard cultural conditions. Beside cryoprotective compounds, the genetic and physiological makeup, the cellular water level and the used method of cryopreservation are also known to determine the success or cells’ regrowth^12^. In *C. roseus*, Mannonen et al.^25^ used sorbitol and DMSO in a two-stage preservation process and successfully conserved *Catharanthus* callus for over 6 months or more without any injury. Cryopreserved cells showed viability, divided well in producing callus colonies in optimized medium. The embryos were similarly differentiated on agar solidified medium at variable numbers like normal embryogenic callus of *C. roseus*. In Pinus and other gymnosperms, where somatic embryogenesis incidence is very frequent, similar embryogenic cultures/axes have been used for successful cryopreservation^34–36^. The present study also investigated the influence of dehydration of varied periods. The highest viability following cryopreservation was observed in 10% PEG after 2 h of dehydration and the maximum mean number of colonies regrown/petri dish was observed in 5% PEG condition. The positive influence of dehydration during cryopreservation was previously noted in other investigated genera and the regenerated plants were reported to be identical to that of mother plants with no morphological variations.

A large number of plants were cryopreserved successfully by using various cryopreservation methods such as in *Capparis spiunosa*^15^, *Artemisia herba-alba*^37^, *Achillea fragrantissima* L.^19^ and many other species^8^. Beside conservation, the ultra-low temperature aims to preserve genetic integrity and the regenerated populations are expected to be the copy of the mother plants^38^. In this case, the regrowth of embryogenic culture, the embryo morphology and the regenerated plants of cryopreserved cells were very similar to the normal embryo-derived plantlet of *C. roseus* with no morphological variation. Ogawa et al.^39^ observed no significant difference in gene expression between cryopreserved and control cells in *Arabidopsis*, when a simple cryopreservation protocol was established in *A. thaliana*, *Daucus carota*, *Lotus japonicus*, *Nicotiana tabacum,* and *Oryza sativa* by using 2 M glycerol, 0.4 M sucrose and 86.9 mM proline solution in protecting cells from freezing damage.

The callus-derived plants show genetic variation and these alterations are caused by several factors like PGRs of various types, aging and culture-induced stresses^40–42^. As these cells met severe cryogenic low-temperature shock/stress and faced several cryoprotective agents, there was a need to evaluate the genetic status of the regenerated plant following cryopreservation. The 2C DNA level of cryopreserved *C. roseus* was estimated and compared with field-grown *Catharanthus* plant. The obtained 2C DNA content of cryopreserved plants is the same and is very consistent with previously reported observations^43,44^. Thus, the cryopreserved regenerated plant's DNA or genome size is unaltered and not affected by adverse low temperature (− 196 °C) stress and cryogenic agents.

Tissue cryopreservation, very similar to seed banking may be useful and is extended to other rare exceptional endangered species; as an alternative, this technology has been explored and applied to a number of rare, endangered plants of flowering taxa, ferns, bryophytes, and algae^38,44,45^. This developed protocol may be used and exploited to other ornamental and economically important plants.

## Materials and methods

### Plant material

*Catharanthus roseus* L. (G). Don was used and cultured as plant material. The plants were collected from the herbal garden of Jamia Hamdard and identified by Prof. Mahendra Sharma, taxonomist, Department of Botany. The identified plant material with voucher specimen (JH-002-98) was preserved in the herbarium and kept in the departmental store/archive room. The various parts of germinated seedlings were used as explants for establishing culture. The process of seed germination and sterilization were reported in earlier communication^46^. The MS^47^ solid and liquid medium were used in which the medium pH was adjusted to 5.8; and the medium sterilization was made at 121 °C for 15 min by using an autoclave. The cultures were kept at 25 ± 2 °C temperature under 16 h photoperiod with cool white fluorescent tubes (100 µmol m^−2^ s^−1^).

### Hypocotyl-embryogenic callus

The calli produced from stem, root and leaf explants were non-embryogenic while the hypocotyl explants induced embryogenic callus on 2,4-D (0.5–1.0 mg/l) supplemented medium; the difference of embryogenic and non-embryogenic tissue and their requirements for plant growth regulators (PGRs) etc. were discussed in detail in earlier work^46^.

### Embryogenic cell suspension culture establishment

Hypocotyl induced embryogenic callus (500 mg) was suspended in conical flasks containing 50 ml of liquid MS medium with 2,4-D (0.5–1.0 mg/l). These suspension containing flasks were sealed and kept on an agitated rotating shaker with an average speed of 120 rpm. The same light regime and photoperiod were provided as described in the earlier section. The suspension was subcultured regularly at an interval of 7 days.

### Embryogenic suspension cells and osmo-protectant treatment

To improve the survivability of embryogenic cells and to increase protection, the suspensions were supplemented with two osmotic agents i.e. sucrose and sorbitol individually. Cell suspension was cultured in MS medium with 2,4-D (0.5–1.0 mg/l) supplemend with sucrose (0.2, 0.4, 0.6 M) or sorbitol (0.2, 0.4, 0.6 M) for 2 days. Control cells were cultured on the same medium without sucrose or sorbitol. The viability and the biomass growth were monitored accordingly.

### Cryoprotectant (CP) treatments

Following treatment with osmotic agents, the suspension cells were placed in cryotubes containing CP agents. The following chemicals and their concentrations were used to evaluate the individual and combined effects of polyethyl glycol (PEG), dimethylsulphonate (DMSO), and glycerol. The level and abbreviation used are CP-A: 5% PEG, CP-B: 10% PEG, CP-C: 5% PEG + 5% DMSO, CP-D: 5% PEG + 10% DMSO, CP-E: 5% PEG + 5% glycerol, CP-F: 5% PEG + 10% glycerol (CP-A to CP-F). The suspension cells were kept in above-mentioned treatments for 1 h and later shifted to a rotating shaker (120 rpm) for one more hour. The viability and regrowth percentages were checked as described below.

### Cryopreservation

Next, the suspension cells of 10 ml each of all groups were poured in 15 ml centrifuge tubes and the cell volume was kept to 20% sedimented cell volume (SCV) by removing the supernatant. The 2.0 ml aliquots of the sedimented cells were taken to a pre-cooled cryotube (− 20 °C) and freezing was done in two steps: first, the cryotubes were kept at − 20 °C for 2 h in a freezer and later transferred to liquid nitrogen (LN) for 1 h.

These cryotubes were withdrawn from LN, thawed immediately at 37 °C in a water bath for 2 min and later added liquid MS medium comprised of 0.2 M sucrose, 1.0 mg/l NAA and 1.5 mg/l BAP for detoxification. These tubes were immediately placed on a rotating shaker at 120 rpm for 24 h at 25 °C temperature. After filtration, the suspended cells were placed on 2,4-D added solid MS medium in Petri dishes to check the viability and formation of colony/callus. The temperature and other cultural conditions are the same as described earlier.

### Viability

The cryopreserved cell viability was tested immediately by MTT [3-(4,5-dimethylthiazole-2-yl)-2,5-diphenyl tetrazolium bromide] assay following Lizbeth et al.^48^ method. Yellow MTT, a tetrazole, and a standard colorimetric assay were used. The purple colour of MTT was measured spectrophotometrically at 570 nm using Lamda Bio 20, UV/VIS spectrophotometer (Perkin Elmer, USA). The percent viability was calculated by using the following formula:

$$ {\text{Viability }}\% = \frac{{\text{MTT value of cryopreserved cells}}}{{\text{MTT value of control}}} \times {1}00 $$

### Regrowth of viable cells after cryopreservation

The viable cells were cultured in 2,4-D (0.5 mg/l) containing solid medium to check the regrowth ability of viable cells. A total of nine Petri dishes were used, the numbers of Petri dishes showing regrowth and the mean numbers of regrown colonies/petridish were counted after 4 weeks of culture.

### The process of dehydration, viability and regrowth of cryopreserved cells

Based on previous experiments’ observation, the best cryoprotective solutions were selected. These select cultures (CP-A and CP-B) were additionally allowed to dehydrate for varied periods (1–5 h) using air under laminar air flow just to observe the influence of decreasing water content on viability. Changes in water content of these select suspension cells were measured hourly. The viability was tested before and after cryopreservation and the best dehydration time was identified. In this experiment, eight Petri dishes were also used; the numbers of Petri dishes showing regrowth was noted and the regrowth percentage was calculated. The mean number of cell colonies per Petri dish was observed after 4 weeks of culture.

### Embryo differentiation and plant regeneration

The numbers of different developing stages ofsomatic embryo (globular, heart, torpedo and cotyledonary) produced from the best two (CP-A and CP-B) cryopreserved regrown cultures were counted and compared with non-frozen control. About 250 mg embryogenic sources of both (frozen and non-frozen) were used, and the number of embryos at different developing stages were counted after 7th week of culture on optimized NAA (0.5 mg/l) and BAP (1.0 mg/l) concentrations. The embryos of cryopreserved culture, similar to non-frozen (control) callus-derived embryos germinated into plantlets later.

### Analysis of 2C DNA content of embryo regenerated plant

Plants developed in vitro from cryopreserved embryogenic culure were taken and the 2C DNA content was measured. The sample was prepared following Galbraith^49^ protocol. The leaf (20 mg) from the embryo regenerated young *C. roseus* shoot and the *Pongamia pinnata* (2C = 2.51 pg DNA), the reference standard were chopped in 0.5 ml Otto I buffer (0.3% citric acid monohydrate, 0.05% NP-40), 50 µg per ml propidium iodide and 100 µg per ml RNase (Sigma-Aldrich USA). The samples were filtered through a 100 µm mesh sieve prior to analyze in CFM BD FACS Calibur (BD Biosciences, San Jose, CA, USA) flow cytometer. The genetic fidelity of the somatic embryo-derived plants was assessed by estimating the 2C DNA content of *C. rosues* by using the following formula and was compared with earlier *C. roseus* report^43^.

$$ {\text{2C DNA content of}}\;C. \, roseus = \,2.51\;{\text{pg}} \times \frac{{ {\text{ Mean position of G}}0/{\text{G1 peak of}}\;C. \, roseus}}{{{\text{Mean position of G}}0/{\text{G1 peak of}}\;P. \, pinnata}} $$

### Vinblastine and vincristine quantification by high pressure thin layer chromatography (HPTLC)

The extraction of leaf tissue and the quantification of vinblastine and vincristine was made following Miura et al.^50^ and Junaid et al.^51^ protocol. About 1.0 gm of leaf tissue of mother plant, kept in vitro and post cryopreserved *C. roseus* plant was dried at 45 °C for about 7 days and was ground. The powder leaf tissues (1.0 g) were shaken in methanol (100%) and extraction was made. The standard curve preparation, the HPTLC instrumentation, and other conditions were discussed earlier^52,53^ and were followed.

### Statistical analyses

All the data on osmotic agents (sucrose or sorbitol), various cryoprotecting agents and their combinations, and dehydration’s effect on viability, vinblastine and vincristine content were expressed as mean ± standard error. For regrowth of embryogenic cultures after cryopreservation without or with dehydration, nine Petri dishes were used; the numbers of Petri dishes showing regrowth was noted, and the regrowth percentage was calculated. In every case, each of the experiments was replicated thrice and was repeated at least twice (n = 6). For flow cytometry, five plants of embryo regenerated origin (five replicates) were used for assessing 2C DNA content. SAS program (Release 9.2, SAS Institute, NC, USA) was used for statistical analysis. Data were subject to the analysis of variance (ANOVA). Duncan’s multiple range test (DMRT) evaluated the differences between the mean values at p ≤ 0.05. The percent values were transformed using arcsine square root (√P) to normalize the error distribution before ANOVA.

### Ethical approval

All procedures were conducted in accordance to the relevant institutional, national, and international guidelines and legislation.

## Conclusions

1. A cryopreservation method of *C. roseus* alkaloid-producing tissues was developed by utilizing osmotica and cryoprotective agents and dehydration.
2. The cryopreserved cells revived, produced embryogenic callus, embryos and plants, very similar to non-cryopreserved embryogenic tissues.
3. The genome size and the 2C DNA of a cryopreserved-derived plant are the same as that of a field-grown *Catharanthus* plant.
4. Therefore, the plant regeneration process, the vincristine, vinblastine yield and the 2C DNA content were not perturbed by adverse ultralow cryogenic temperature and agents.

## Data Availability

The authors certify that all the data of this study are available within this manuscript.
